# Cost-Reference Particle Filter for Cognitive Radar Tracking Systems with Unknown Statistics

**DOI:** 10.3390/s20133669

**Published:** 2020-06-30

**Authors:** Lei Zhong, Yong Li, Wei Cheng, Yi Zheng

**Affiliations:** School of Electronics and Information, Northwestern Polytechnical University, Xi’an 710072, China; zhonglei@mail.nwpu.edu.cn (L.Z.); pupil_119@nwpu.edu.cn (W.C.); cuzgen@mail.nwpu.edu.cn (Y.Z.)

**Keywords:** cognitive radar, particle filter, target tracking, bayesian bounds, nonlinear model

## Abstract

A novel robust particle filtering algorithm is proposed for updating both the waveform and noise parameter for tracking accuracy simultaneously and adaptively. The approach is a significant step for cognitive radar towards more robust tracking in random dynamic systems with unknown statistics. Meanwhile, as an intelligent sensor, it would be most desirable for cognitive radar to develop the application of a traditional filter to be adaptive and to expand the adaptation to a wider scope. In this paper, after analysis of the Bayesian bounds and the corresponding cost function design, we propose the cognitive radar tracking method based on a particle filter by completely reconstructing the propagation and the update process with a cognitive structure. Moreover, we develop the cost-reference particle filter based on optimizing the cost function design according to the complicated system or environment with unknown statistics. With this method, the update of the estimation cost and variance arrives at the approximate optimization, and the estimation error can be more adjacent to corresponding low bounds. Simulations about the tracking implementation in unknown noise are utilized to demonstrate the superiority of the proposed algorithm to the existing methods in traditional radar.

## 1. Introduction

### 1.1. Problem Statement

Cognitive radar can basically be defined as: an intelligent radar system of hardware and software in which the transmit and receive parameters (i.e., pulse length, pulse repetition frequency (PRF), modulation, power, frequency, and polarization) are selected, in real-time, and use adaptation between the information extracted from the sensor/processor and the design and transmission of subsequent waveforms, in response to the observed scene to optimize the performance of a given application. The problem of target tracking in cognitive radar system has received considerable attention. It is well known that most of the state-space dynamical systems are nonlinear or non-Gaussian. For example, the multi-state transition of a drone from hovering to maneuvering is often nonlinear, and the measurement noise is mainly flicker noise or heavy-tailed noise [1].

Despite the performance decline of extended Kalman filter (EKF) and unscented Kalman filter (UKF) in highly nonlinear problems, and that general analytical solutions are intractable in nonlinear or non-Gaussian systems, solutions continue to emerge from different viewpoints. However, most of the methods rely on the assumption that the noise has known statistics, or they require accurate mathematical representation of the dynamics of the system evolution; otherwise, it is almost impossible to directly approximate the true distribution. In practice, sometimes the assumption is in accordance with the actual situation, but sometimes not. The same situation occurs in cognitive radar systems. Without explicit mathematical models or a priori information, how cognitive radar can still exert its advantages is a concern of this article.

We briefly review various existing methods to cognitive radar tracking problem that involve particle filter (PF) in some relevant manner.

### 1.2. Related Works

For handling arbitrary nonlinear models and arbitrary noise distributions, methods based on Monte Carlo (MC) methodology have emerged in Bayesian estimation. MC is a simulation-based method aimed at estimating the posteriori pdf of the state given the observations. Markov Chain Monte Carlo (MCMC) and Sequential MC (SMC) are two main tools in it, and they can sample from high dimensional probability distributions. The performance of MCMC would be unreliable when the proposal distributions that are used to explore the space are poorly chosen and/or if highly correlated variables are updated independently. While, PF became popular for it is particularly suitable for real-time estimation. 

The Sequential Importance Sampling (SIS) algorithm was used for the first time to solve the problem of nonlinear filtering [2]. The formal establishment of PF was attributed to the proposal of resampling technology [3,4]. Meanwhile, the idea of Sampling Importance Resampling (SIR) was discovered and developed [5]. PF is allowed to express a complete and precise state posterior distribution, so any statistical data such as mean, variance, and modulus can be easily calculated, and theoretically, the accuracy is higher than that of EKF and UKF in nonlinear systems [6]. Hence, despite its computational pressure, it is still quite attractive to us. During the development of PF, there are several inevitable drawbacks need to be addressed: (1) How to approximate the optimal proposal distribution further; (2) How to overcome the problems of weight degeneracy, sample impoverishment, etc., to make the resampling more effective; (3) How to make the algorithm efficient and online. 

Therefore, more variants of PF were produced [7]. Efficient importance sampling techniques were studied in [8,9] for the first problem, but these algorithms require that the posterior distribution of the states are assumed as a priori known and can be approximated by a Gaussian distribution. An auxiliary variable particle filter was used to deal with the second problem [10], but the filtering performance degrades when the state noise is strong, and because the likelihood function and weight value need to be calculated twice for each particle, the calculation amount increases. Adaptive PF (APF) can release the computation burden by adjusting the number of particles dynamically [11,12,13,14,15]. This method chooses a small particle number if the density is focused on a small part of the state-space, and chooses a large number if the state uncertainty is high [16]. KLD-APF is based on Kullback–Leibler (KL) information or KL distance (KLD) sampling, but it ignores any mismatch between the true and the proposal distribution [17]. When the mismatch happens, the addition of particle number will only increase the computational load. On the other hand, reducing the particle number may aggravate the sample impoverishment and further weaken the effect of resampling.

In addition to these drawbacks, PF would still be invalid when some issues are encountered. PF requires that the conditional pdf of the observed variable can be estimated, otherwise the weight of the particles cannot be calculated. Thus, particle MCMC (PMCMC) was proposed by combing SMC with MCMC as an efficient approach [18]. Based on PMCMC, the SMC^2^ algorithm is motivated to tackle the intractable problem of probable increments in state-space models [19]. Similar to the SMC^2^ scheme, nested particle filters for online parameter estimation were proposed but in a purely recursive manner, in order to address the problem of approximating the posterior probability distribution of the fixed parameters of a state-space dynamical system [20]. On robustness, cooperative parallel particle filters were designed for the dual purpose of Bayesian inference and on-Line Model Selection, with the online adaptation for the particle number [21]. Particle learning was proved in [22] to be outperforming existing PF alternatives and a competitor to MCMC. In the presence of model uncertainty where discrete data are encountered, a new SMC method was proposed for the filtering and prediction of time-varying signals [23]. A similar method with better predictive powers was proposed in [24], wherein the resampling step could be dynamically adjusted and the predictive powers could be updated sequentially as more data were observed. Moreover, what deserves attention is that Bayes is not the only choice, as a neural filter based on GRNN is also outperforming numerical filters in state vector estimation during dynamic changes of target movement parameters [25].

Inspired by the previous research, we might find a solution independent of the prior information through updating the proposal distribution and its stepwise approximation to the true distribution by iterative methods. The authors of [26] designed a cost function in PF to iteratively update the state and variance when the prior information is unknown. Naturally, it has been applied to many areas such as target tracking, autonomous vehicle positioning, sensor network, orthogonal frequency division multiplexing (OFDM) systems, wireless local area network (WLAN), and tilt estimation [27,28,29]. There are some improvements to the algorithm. In [30], a particle selection algorithm was proposed and analyzed for implementation with parallel computing devices and to circumvent the main drawback of the conventional resampling techniques. Authors of [31] melded random measures of two or more cost-reference particle filters to obtain a fused random measure that combines the information from the individual cost-reference particle filters. However, there is no further study on the optimization of the cost function, especially when the tracking structure can be adaptive. Cognitive radar designs the optimal estimator for Bayesian framework in [32], but the majority of the existing Bayesian tracking methods for cognitive radar applications are based on the Kalman filter for linear systems [33,34], and Kalman-like solutions, e.g., cubature KF (CKF) and continuous-discrete (CD)-CKF for nonlinear problems [35]. Few researches use PF. In [36], a particle filter combined with probabilistic data association is used as a tracker. In [37], a cognitive structure was designed as only a part of PF, namely, a parallel structure of PF, while EKF was used by waveform selection to adapt the particle number and reduce the computation cost.

### 1.3. Contributions and Organization of the Paper

When we specify PF as the nonlinear tracking method in a cognitive radar, we consider using the cognitive structure to expand the state-space of PF or its variants to another dimension where the waveform parameters can be changed from fixed to dynamic, and we can optimize the cost function design and corresponding lower bound of the estimation error. In turn, using the particle filter tracking method based on the optimal cost function can expand the scope of the application of cognitive radar. The main contributions of this paper will be presented by the following items:

(1) The cognitive radar tracking method based on PF is proposed and the mathematical model of which it is derived for the first time. Push the cognitive radar tracking framework from existing the Kalman-like to the developing SMC-like. Not only the data process but also the waveform design is in the PF iteration, that is, a fully cognitive PF.

(2) Refine the idea of cost-reference PF with cognitive framework. When the estimation of the parameters (i.e., signal-to-noise ratio (SNR)) in cognitive radar mismatches the real situation, a novel cognitive cost-reference particle filter algorithm is proposed to bring about robustness to the existing cognitive radar tracking methods and intelligence for the current adaptive method. The convergence is proofed mathematically. 

(3) The Cramér–Rao Lower Bound (CRLB) of the proposed cognitive PF is derived, and the corresponding cost function is designed. 

The rest of this paper is organized as follows: the standard PF, the mathematical model of cognitive radar, and the interface of PF to cognitive algorithm are presented, and the CRLB of the cognitive PF is derived in Section 2. The principle of the cost-reference particle filter is restated, the proposed cognitive scheme is presented along with the implementation steps, and the convergence of the solution is proofed in Section 3. Section 4 shows the dynamic model, the maneuvering target tracking in unknown non-Gaussian noise, and the simulation results. Section 5 presents the conclusions.

## 2. PF for Cognitive Radar Tracking 

In this section, the cognitive tracking method based on PF is formed to address the non-linear and non-Gaussian state estimation problem in cognitive radar. 

### 2.1. PF and Cognitive Radar Model

#### 2.1.1. Standard PF 

The basic idea is to generate a set of random samples in the state-space according to the empirical conditioning distribution of the system state vector. They are called particles, the weight and position of which are continuously adjusted according to the measurement, and the initial empirical conditioning distribution is modified. The MC estimate of the integral can be written as:

(1) E[f(x)]=∫f(x)p(x)dx=∫f(x)[p(x)/q(x)]q(x)dx.

Let $\omega \left(x_{}^{\left(i\right)}\right)=p\left(x_{}^{\left(i\right)}\right)/q\left(x_{}^{\left(i\right)}\right)$ denote the importance weights. Equation (1) is calculated by generating N≫1 independent samples $\left\{x_{}^{\left(i\right)}:i=1,\ldots ,N\right\}$ distributed according to q(x). The weighted sum is formed and normalized as:

(2) $$E_{N}\left[f\left(x\right)\right]=\frac{1}{N}\sum_{i=1}^{N}f\left(x_{}^{\left(i\right)}\right)\omega \left(x_{}^{\left(i\right)}\right),$$

(3) $$E\left[f\left(x\right)\right]=\frac{\frac{1}{N}\sum_{i=1}^{N}f\left(x_{}^{\left(i\right)}\right)\omega \left(x_{}^{\left(i\right)}\right)}{\frac{1}{N}\sum_{j=1}^{N}\omega \left(x_{}^{\left(j\right)}\right)}=\sum_{i=1}^{N}f\left(x_{}^{\left(i\right)}\right)\tilde{\omega }\left(x_{}^{\left(i\right)}\right).$$

The weight update equation can be shown to be:

(4) $$\omega \left(x_{k}^{\left(i\right)}\right)=\omega \left(x_{k-1}^{\left(i\right)}\right)\frac{p\left(z_{k}^{}|x_{k}^{\left(i\right)}\right)p\left(x_{k}^{\left(i\right)}|x_{k-1}^{\left(i\right)}\right)}{q\left(x_{k}^{\left(i\right)}|x_{k-1}^{\left(i\right)}\right)}.$$

Substitution of $q\left(x_{k}^{\left(i\right)}|x_{k-1}^{\left(i\right)}\right)=p\left(x_{k}^{\left(i\right)}|x_{k-1}^{\left(i\right)}\right)$ into Equation (4) yields:

(5) $$\omega \left(x_{k}^{\left(i\right)}\right)=\omega \left(x_{k-1}^{\left(i\right)}\right)p\left(z_{k}^{}|x_{k}^{\left(i\right)}\right).$$

When N→∞, the posterior probability density can be approximated by the following:

(6) $$p\left(x_{k}^{}|z_{1:k}^{}\right)\approx \sum_{i=1}^{N}\omega \left(x_{k}^{\left(i\right)}\right)\delta \left(x_{k}^{}-x_{k}^{\left(i\right)}\right),$$

where δ(⋅) denotes the Dirac delta measure, with defining properties: (1) $\delta \left(x_{k}^{}-x_{k}^{\left(i\right)}\right)=0$ for $x_{k}^{}\neq x_{k}^{\left(i\right)}$, (2) $\delta \left(x_{k}^{}-x_{k}^{\left(i\right)}\right)=+\infty$ for $x_{k}^{}=x_{k}^{\left(i\right)}$; and (3) $\int_{-\infty }^{\infty }\delta \left(x_{k}^{}-x_{k}^{\left(i\right)}\right)dx_{k}^{}=1$. More details about the SIR-PF can refer to [38]. It has been termed standard PF (SPF) in the simulation below. The accuracy approximates the optimal estimation.

#### 2.1.2. Cognitive Radar System Model

The radar transmitter through the emission exploiting waveform selection stimulates the background with the goal to obtain a response from it such as a radar echo. The mentioned response is perceived by radar receiver, which plays the equivalent role of the human senses. The detector performs a low-level processing of received sensor data. The scene analyzer is conceived by exploiting previously acquired information to estimate the statistical characteristics of the operating environment. The tracker acts as a high-level processor.

The functions manager receives requests for radar activities (e.g., search, tracking) from the keyboard according to a specific mode of working or operator requirements. The functional interaction is with the radar hardware, the signal processor, the data processor, and the external peripherals. Starting from the state estimate ${\hat{x}}_{k-1}^{}$ and state error covariance at time step k − 1, the transmitter uses waveform parameter $\theta_{k-1}$ to illuminate the environment. The receiver performs a prediction and obtains ${\hat{x}}_{k}^{\left(i\right)}$ (i.e., Equation (49)) with the previous value and current measurement. Then, ${\hat{x}}_{k}^{\left(i\right)}$ is utilized to evaluate the CRLB, and thus, the measurement error covariance matrix $R_{k}\left(\theta \right)$. For each waveform of the library, the predicted error covariance matrix $P_{k}$ is computed for evaluation (i.e., Equation (5) in Algorithm 1). At this point, a cost function (i.e., Equation (18)) is to be formulated, and the waveform at the next transmission is chosen as the one that minimizes the weighted mean square error (MSE) on the target state, namely minimizing the cost function by the optimal waveform design or parameters adjustment. The transmitted signal then provides a new optimized measurement $z_{k}$ to feed the PF processor and track the target. The obtained target state estimate ${\hat{x}}_{k}^{}$ is finally exploited to predict the next target state ${\hat{x}}_{k+1}^{\left(i\right)}$, and optimizes the new transmitted waveform according to the closed-loop paradigm. This constructs the PAC in cognitive radar [39].

**Figure 1:**
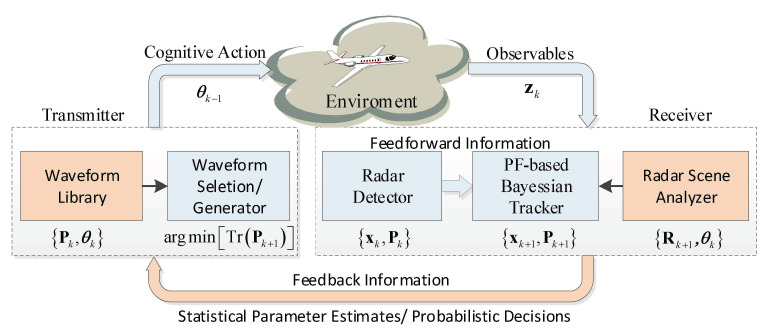
Cognitive radar system for target tracking.

Summarizing, the block scheme of a cognitive tracking system architecture and the information-flow in the mathematical model is displayed in Figure 1. Notice that, if the blocks in red are cancelled, the rest blocks in blue degrade to the traditional radar system.

### 2.2. PF-Based Cognitive Tracking Algorithm

#### 2.2.1. Bayesian Bounds for Cognitive Radar

The recursive Bayesian estimators, such as the EKF or PF, work in the non-linear system employing the feed-forward processing chain, and do not consider the feedback information from the receiver to the transmission of subsequent waveforms. So far, the corresponding recursive BCRB on the MSE matrix of the state vector was derived and presented in [40]. It can be obtained by calculating the Fisher information matrix (FIM) [41], the inverse of which is the unbiased estimator of the CRLB of the state estimation error covariance [42]. In this paper, the PF based on the cognitive structure characterized by feedback is analyzed, and the estimation is biased. Bayesian FIM (BIM) for this case can be developed and computed iteratively using this form [43]:

(7) $$B_{k}^{↑}\left(\theta_{k}|z_{1:k-1};\Theta_{k-1}\right)=B_{k}^{-}\left(\theta_{k}|z_{1:k-1};\Theta_{k-1}\right)+J_{k}^{-}\left(\theta_{k}|z_{1:k-1};\Theta_{k-1}\right),$$

where $B_{k}^{-}\left(\theta_{k}|z_{1:k-1};\Theta_{k-1}\right)$ represents the contribution of the prior information is prior, called the prior term, and is denoted as $J_{P}$; the $J_{k}^{-}\left(\theta_{k}|z_{1:k-1}^{};\Theta_{k-1}\right)$ represents the contribution of the data, called data term and denoted as $J_{D}$.

(8) $$J_{D}=E_{k}\left\{J_{F}\left(x_{k}^{}\right)\right\}=E_{k}\left\{-E_{z_{k}^{}|x_{k}^{};\theta_{k}}\left\{\frac{\partial^{2}\ln f\left(z_{k}^{}|x_{k}^{};\theta_{k}\right)}{\partial x_{k}^{2}}\right\}\right\},$$

(9) $$J_{P}=-E_{x_{k}^{}}\left\{\frac{\partial^{2}\ln f\left(x_{k}^{}\right)}{\partial x_{k}^{2}}\right\}.$$

The lower bound is defined as the inverse of the BIM, as the form:

(10) $$BCRB={\left[J_{D}+J_{P}\right]}^{-1}.$$

The lower bound of CR would be smaller than the general BCRB, which can be proved as:

(11) $${\left[J_{D}\left(\theta_{k}\right)+J_{P}\left(\theta_{k}\right)\right]}^{-1}\leq {\left[J_{D}+J_{P}\right]}^{-1}=BCRB.$$

#### 2.2.2. Cost Function Design

As we know, CRLB is the possible MMSE of an unbiased estimation. It can be proved that minimizing the trace of the covariance is equal to the MMSE of the expectation of tracking state [38]; thus, we choose the minimum trace with respect to the waveform parameters as the processor cost function modeled by:

(12) $$C_{\Theta }\left(\theta_{k}|z_{1:k-1}^{};\Theta_{k-1}\right)=E_{x_{k}\;z_{k}|I_{k}\;\theta_{k}}\left[{\left(x_{k}-{\hat{x}}_{k}\left(z_{k}^{}\right)\right)}^{T}\left(x_{k}-{\hat{x}}_{k}\left(z_{k}^{}\right)\right)\right]\approx \mathrm{tr}\left\{B_{k}^{↑}{\left(\theta_{k}|z_{1:k-1}^{};\Theta_{k-1}\right)}^{-1}\right\},$$

where tr(⋅) is an operator that extracts the trace of $B_{k}^{↑}{\left(\theta_{k}|z_{1:k-1}^{};\Theta_{k-1}\right)}^{-1}$, and the waveform parameters are denoted as $\Theta \equiv \left\{\theta_{1},\theta_{2},\ldots ,\theta_{k}\right\}$. ${\hat{x}}_{k+1}\left(I_{k},x_{k+1},z_{k+1};\theta_{k}\right)$ is the posterior predictive value of the state estimation given waveform parameter. Since ${\hat{x}}_{k+1}$ is related to $z_{k+1}$, which is a nonlinear mapping of $x_{k+1}$, we use approximation rather than integration to solve this expectation. $C_{\Theta }\left(\theta_{k}|z_{1:k-1}^{};\Theta_{k-1}\right)$ is approximated by $\mathrm{tr}\left\{B_{k}^{↑}{\left(\theta_{k}|z_{1:k-1}^{};\Theta_{k-1}\right)}^{-1}\right\}$ [33,35]. The model of $P_{k+1|k+1}$ in KF can refer to [44], while in PF, $P_{k+1|k+1}$ is modeled by a different form [38]. More details of the relation between $P_{k+1|k+1}$ and $R_{k}\left(\theta_{k-1}\right)$ are referred to in [35]. We derive the model of measurement noise covariance based on waveform selection as [45]:

(13) $$R_{k}\left(\theta_{k-1}\right)=\Gamma B_{k}^{↑}{\left(\theta_{k}|z_{1:k-1}^{};\Theta_{k-1}\right)}^{-1}\Gamma =\Gamma U^{-1}\left(\theta_{k-1}\right)\Gamma /\eta ,$$

(14) $$\Gamma ≜\mathrm{diag}\left[\frac{c}{2},\frac{c}{4\pi f_{c}}\right],$$

where $\eta =2E_{R}/N_{0}$ is the SNR, and $N_{0}$ denotes the spectral density of the complex noise envelope $\tilde{n}\left(k\right)$. $U\left(\theta_{k-1}\right)$ is a scaled version of FIM. Γ denotes a symmetric matrix defined as this form. c is the speed of waveform propagation, and $f_{c}$ is the carrier frequency.

When the LFM signal with Gaussian amplitude modulation is selected as the transmit waveform, with the duration of Gaussian pulse *λ* and the chirp rate *b* chosen to form the parameter vector $\theta_{k-1}=\left[\lambda ,b\right]$, the measurement noise covariance matrix can be modeled by $R_{k,r\dot{r}}\left(\theta_{k-1}\right)$ [41]:

(15) $$R_{k}\left(\theta_{k-1}\right)=R_{k,r\dot{r}}\left(\theta_{k-1}\right)=\left[\begin{array}{cc} \frac{c^{2}\lambda^{2}}{2\eta } & -\frac{c^{2}b\lambda^{2}}{2\pi f_{c}\eta }\\ -\frac{c^{2}b\lambda^{2}}{2\pi f_{c}\eta } & \frac{c^{2}}{{\left(2\pi f_{c}\right)}^{2}\eta }\left(\frac{1}{2\lambda^{2}}+2b^{2}\lambda^{2}\right) \end{array}\right].$$

The sensor cost function is defined as:

(16) $$C_{\Theta }\left(\theta_{k}\right)=\left\{ \begin{array}{ll} 0 & \theta_{k}\in \Theta \\ \infty & \mathrm{otherwise} \end{array}\right..$$

The loss function is defined with respective to the cost function of the processor and sensor, modeled by:

(17) $$L_{C,\Theta }\left(\theta_{k}|z_{1:k-1}^{};\Theta_{k-1}\right)\equiv C_{\Theta }\left(\theta_{k}|z_{1:k-1}^{};\Theta_{k-1}\right)+C_{\Theta }\left(\theta_{k}\right).$$

The choice of the optimal parameter is equivalent to the optimization problem given by:

(18) $$\theta_{k}=\underset{\theta_{k}^{}\in P}{\arg\min}C_{\Theta }\left(\theta |z_{1:k-1}^{};\Theta_{k-1}\right)\;s.t.\;\theta \in \Theta .$$

#### 2.2.3. Algorithm and Numerical Simulation

We have restated the principle of the cognitive radar system, and have given the interface from PF to it to induce the human cognition mechanism into the PF algorithm. As with the statements before, SPF is available to be the tracking method of the cognitive radar for a tracking target with a nonlinear dynamic and non-Gaussian distribution. The basic algorithm steps of SPF along with the interface to cognitive tracking framework is given as follows Algorithm 1:
**Algorithm 1.** Cognitive Radar Tracking Recursion Based on PF**Initialization** 1.    ${\hat{x}}_{0}^{\left(i\right)}∼p\left(x_{0}\right)$, $\omega_{k}^{\left(i\right)}=1/N_{s}$, $i=1,\ldots ,N_{s}$
**Controller Optimization** 2.    ${\hat{x}}_{k}^{\left(i\right)}∼q\left(x_{k}^{\left(i\right)}|{\hat{x}}_{k-1}^{\left(i\right)},z_{k}\right)$, the mean $E_{p_{k}\left(x_{k}^{}|{\hat{x}}_{k-1}^{\left(i\right)}\right)}\left[{\hat{x}}_{k}^{}\right]=f_{x}\left({\hat{x}}_{k-1}^{\left(i\right)}\right)$, $i=1,\ldots ,N_{s}$
 3.    $\omega_{k}^{\left(i\right)}\left(\theta \right)\propto p\left(z_{k}|{\hat{x}}_{k}^{\left(i\right)}\right)p\left({\hat{x}}_{k}^{\left(i\right)}|x_{k-1}^{\left(i\right)}\right)/q\left(x_{k}^{\left(i\right)}|x_{0:k}^{\left(i\right)},z_{1:k}\right)$
$,\;i=1,\ldots ,N_{s}$
$,\;\mathrm{normalize}\;{\tilde{\omega }}_{k}^{\left(i\right)}\left(\theta \right)=\omega_{k}\left(x_{0:k}^{\left(i\right)}\right)/\sum_{i=1}^{N_{s}}\omega_{k}\left(x_{0:k}^{\left(i\right)}\right)$
 4.    $\mathrm{If}\;N_{eff}=1/\sum_{i=1}^{N_{s}}{\left({\tilde{\omega }}_{k}^{\left(i\right)}\right)}^{2}<N_{th}\left(\mathrm{empirical}\right)$
$,\;\mathrm{then}\;\left[{\left\{{\hat{x}}_{k}^{\left(i\right)},{\hat{\omega }}_{k}^{\left(i\right)}\left(\theta \right)\right\}}_{i=1}^{N_{s}}\right]=\mathrm{RESAMPLE}\left[{\left\{x_{k}^{\left(i\right)},{\tilde{\omega }}_{k}^{\left(i\right)}\left(\theta \right)\right\}}_{i=1}^{N_{s}}\right]$
 5.    $P_{k}\left(\theta \right)=\sum_{i=1}^{N_{s}}\omega_{k}^{\left(i\right)}\left(\theta \right)\left({\hat{x}}_{k}^{\left(i\right)}-{\hat{x}}_{k}^{}\right){\left({\hat{x}}_{k}^{\left(i\right)}-{\hat{x}}_{k}^{}\right)}^{T}$
 6.    $\theta_{k}^{*}=\arg\underset{\theta_{k}^{}\in P}{\min}\left[\mathrm{Tr}\left({\bar{P}}_{k+1|k+1}^{}\left(\theta_{k}\right)\right)\right]$
$.\;\mathrm{Select}\;\mathrm{the}\;\mathrm{optimal}\;\theta_{k}=\theta_{k}^{*}$
**Motion Update and Measurement** 7.    $f^{-}\left({\hat{x}}_{k-1}^{\left(i\right)}\right)=\int q\left(x_{k}^{\left(i\right)}|x_{k-1}^{\left(i\right)};\theta_{k}\right)f\left({\hat{x}}_{k-1}^{\left(i\right)}\right)dx_{k-1}^{}$$,\;{\hat{x}}_{k-1}^{\left(i\right)}=\mu_{k}^{-}=E_{k}^{-}\left[{\hat{x}}_{k}^{}\right]$ 8.    ${\hat{y}}_{k}=\arg\underset{y}{\min}\ln f\left(z_{k}^{}|y;\theta_{k}\right)$
**Information Update and State Estimation** 9.    $\left\{{\hat{x}}_{k}^{\left(i\right)},\omega_{k}^{\left(i\right)}\right\}=\left\{x_{k}^{\left(i\right)},{\tilde{\omega }}_{k}^{\left(i\right)};\theta_{k}\right\}$
$,\;i=1,\ldots ,N_{s}$
$,\;{\hat{x}}_{k}\approx \sum_{i=1}^{N_{s}}\omega_{k}^{\left(i\right)}{\hat{x}}_{k}^{\left(i\right)}$


As an illustration of the CRLB, consider the scenario that the target is assumed to move with a constant velocity along a straight line in the Cartesian plane. The target kinematic state (position, velocity) ${\left[x_{1,k}^{},x_{2,k}^{},x_{3,k}^{},x_{4,k}^{}\right]}^{T}$ is estimated from the noise-corrupted measurements $z_{k}={\left[r_{k},\;\beta_{k}\right]}^{T}+w_{k}$ with $r_{k}={\left(x_{1,k}^{2}+x_{2,k}^{2}\right)}^{1/2}$, $\beta_{k}=\arctan\left(x_{2,k}^{}/x_{1,k}^{}\right)$. Thus, the measurement equation is nonlinear. The measurement noise $w_{k}$ is assumed to be white, zero-mean Gaussian, with covariance $R=diag\left[\sigma_{r}^{2},\sigma_{\beta }^{2}\right]$, and with the sampling interval T = 0.1 s. The covariance of the process noise is $Q=\sigma_{x}^{2}\cdot diag\left[T_{0},T_{0}\right]$, wherein $T_{0}=[0.5T^{4}\;0.5T^{3};\;0.5T^{3}\;T^{2}]$. The initial true state of the target is ${\left[4000,\;80,\;12,000,\;-20\right]}^{T}$, and the given initial state is set as ${\left[5000,\;90,\;15,000,\;-20\right]}^{T}$, with an estimation error covariance of $P_{0}=1000I$.

**Figure 2:**
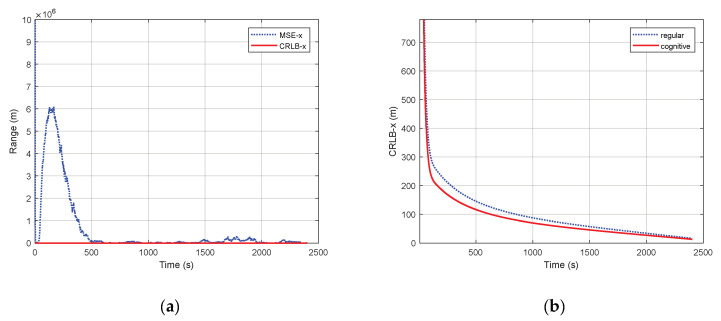
Mean square error (MSE) and posterior Cramér–Rao bound (PCRB): (**a**) Comparison of PCRB and MSE; (**b**) Comparison of PCRB with fixed waveform and dynamic waveform, position $x_{k}$.

Figure 2 shows the results in terms of posterior Cramér–Rao bound (PCRB) and root mean square errors (RMSEs) in the range estimation. As observed in Figure 2a, when we perform the proposed CPF for target tracking in cognitive radar systems, the MSE is initially larger than PCRB, due to the initialization that is not exactly matched to PCRB. Very soon though, CPF demonstrates a fast convergence. Then, MSE agrees with the theoretical curve for PCRB, presenting a considerably well overall performance. Figure 2b compare the PCRB curves obtained using a fixed waveform in a traditional radar and dynamic waveform in cognitive radar. Obviously, the PCRB in the cognitive radar provides a valid lower bound for all time steps, and the convergence is faster.

As we know, the CRLB does not care about the estimation method, but only reflects the best effect of using the existing available information to estimate the parameters. Thus, it can be seen from the figures that after the cognitive system utilizes the extra environmental information through the closed-loop structure, it is possible to lower the previous bounds further.

## 3. Cognitive Tracking Problem Model in Unknown Environment

In this section, we develop the cost-reference PF (CRPF) for a cognitive robust filter. This means that we extend the classic cognitive radar scenarios to an unknown environment. Specifically, the general description of the CRPF approach is listed first, then the details on fusing the cost with cognitive method are specified.

### 3.1. Cost-Reference Particle Filtering Approach 

As we all know, SPF works well when the mathematical forms of the probability distributions of the noise are assumed explicit, but when the mathematical representation of the dynamics of the system evolution is unknown a priori, or the assumptions of probabilistic models cannot be achieved, SPF may fail and the estimation results may be inaccurate even if they have the cognitive structure.

Unlike basic PF, which only gives the state vectors in sequential algorithm, CRPF gives the state samples and associated costs, namely the WPS as the form:

(19) $$\Xi_{k}={\left\{x_{k}^{\left(i\right)},C_{k}^{\left(i\right)}\right\}}_{i=1}^{M},$$

where $C_{k}^{\left(i\right)}=C\left(x_{0:k}^{\left(i\right)}|y_{1:k}^{},\lambda \right)$ is the cost of the particle $x_{k}^{\left(i\right)}$. The cost function can be denoted by a recursive additive structure:

(20) $$C\left(x_{0:k}^{}|z_{1:k}^{},\lambda \right)=\lambda C\left(x_{0:k-1}^{}|z_{1:k-1}^{},\lambda \right)+\Delta C\left(x_{k}^{}|z_{k}^{}\right),$$

where λ is a forgetting factor, which is used to avoid attributing an excessive weight to old observations. $\Delta C\left(x_{k}^{}|z_{k}^{}\right)$ is the incremental cost function, the prediction of which can be obtained as:

(21) $$\Delta C\left(f_{x}\left(x_{k-1}^{}\right)|z_{k}^{}\right)=R\left(x_{k-1}^{}|z_{k}^{}\right),$$

where the one-step risk function is introduced, and the risk of particle i can be computed as:

(22) $$R:R^{L_{x}}\times R^{L_{y}}\to R,\;x_{k-1}^{},z_{k}^{}\to R\left(x_{k-1}^{}|z_{k}^{}\right),$$

(23) $$R_{k+1}^{\left(i\right)}=\lambda C_{k}^{\left(i\right)}+R\left(x_{k}^{\left(i\right)}|z_{k+1}^{}\right).$$

We assign the particles in $\Xi_{k+1}$ the PMF, which is, according to the cost, defined as:

(24) $$\pi_{k+1}^{\left(i\right)}\propto \mu \left(C_{k+1}^{\left(i\right)}\right),$$

where a monotonically decreasing function can be used as μ(⋅), which is selected to guarantee an adequate discrimination of low-cost particles from higher ones. Then, we would obtain the minimum cost by maximum the function below:

(25) $${\tilde{x}}_{0:k+1}^{\min}=x_{k+1}^{\left(i_{0}\right)},i_{0}=\arg\underset{i}{\max}\left\{\pi_{k+1}^{\left(i\right)}\right\}.$$

Notice that the mean value here is equal to the minimum cost estimate, and it even has slight advantages over the latter one. More details can be referred to in [26], and the presentation can be found in Algorithm 2.

The overall procedure of the CRPF algorithm is summarized in Algorithm 2.
**Algorithm 2.** CRPF algorithm for target tracking problem**Initialization** 1.    $x_{0}^{\left(i\right)}∼p_{0}\left(x_{0}\right)$
$,\;C_{0}^{\left(i\right)}=0$
$,\;\sigma_{0}^{2,\left(i\right)}$
$,\;i=1,\ldots ,N_{s}$
$,\;\mathrm{the}\;\mathrm{weighted}\text{-}\mathrm{particle}\;\mathrm{set}\;\Xi_{0}={\left\{x_{0}^{\left(i\right)},C_{0}^{\left(i\right)}\right\}}_{i=1}^{N_{s}}$
**PMF Update** 2.    $R_{k}^{\left(i\right)}=\lambda C_{k-1}^{\left(i\right)}+{\left\|z_{k}-f_{y}\left(f_{x}\left(x_{k-1}^{\left(i\right)}\right)\right)\right\|}^{q}$
, q=1,2
$,\;{\hat{\pi }}_{k}^{\left(i\right)}\propto \mu \left(R_{k}^{\left(i\right)}\right)=\frac{1}{{\left(R_{k}^{\left(i\right)}-\min{\left\{R_{k}^{\left(i\right)}\right\}}_{i=1}^{N_{s}}+\delta \right)}^{\beta }}$
$,\;i=1,\ldots ,N_{s}.$
 3.    ${\left\{{\hat{x}}_{k-1}^{\left(i\right)},{\hat{C}}_{k-1}^{\left(i\right)}\right\}}_{i=1}^{N_{s}}=\mathrm{RESAMPLE}\left[{\left\{{\hat{\pi }}_{k}^{\left(i\right)}\right\}}_{i=1}^{N_{s}}\right]$
**Particle Propagation and Variance Update** 4.    $E_{p_{k}\left(x_{k}^{}|{\hat{x}}_{k-1}^{\left(i\right)}\right)}\left[x_{k}^{}\right]=f_{x}\left({\hat{x}}_{k-1}^{\left(i\right)}\right)$
$,\;Cov_{p_{k}\left(x_{k}^{}|{\hat{x}}_{k-1}^{\left(i\right)}\right)}\left[x_{k}^{}\right]=\sigma_{k}^{2,\left(i\right)}I_{\left[x\right]}$
$,\;x_{k}^{\left(i\right)}∼p_{k}\left(x_{k}^{}|{\hat{x}}_{k-1}^{\left(i\right)}\right).$
 5.    $\sigma_{k}^{2,\left(i\right)}=\left\{ \begin{array}{ll} \sigma_{k-1}^{2,\left(i\right)} & t\leq 10\\ \frac{k-1}{k}\sigma_{k-1}^{2,\left(i\right)}+\frac{{\left\|x_{k}^{\left(i\right)}-g\left({\hat{x}}_{k-1}^{\left(i\right)}\right)\right\|}^{2}}{k\times \dim\left[x\right]} & t>10 \end{array}\right.$
$,\;i=1,\ldots ,N_{s}$
**State Estimation** 6.    $C_{k}^{\left(i\right)}=\lambda C_{k-1}^{\left(i\right)}+{\left\|z_{k}-f_{y}\left(x_{k}^{\left(i\right)}\right)\right\|}^{q}$
$,\;{\hat{\pi }}_{k}^{\left(i\right)}\propto \mu_{2}\left(C_{k}^{\left(i\right)}\right)=\frac{1}{{\left(C_{k}^{\left(i\right)}-\min{\left\{C_{k}^{\left(i\right)}\right\}}_{i=1}^{N_{s}}+\delta \right)}^{\beta }}$
, δ,β>0
$,\;\mathrm{normalized}\;\mathrm{to}\;\pi_{k}^{\left(i\right)},$
 7.    ${\hat{x}}_{k}=x_{k}^{mean}=\sum_{i=1}^{N_{s}}\pi_{k}^{\left(i\right)}x_{k}^{\left(i\right)}$
$,\;i=1,\ldots ,N_{s}$


### 3.2. Cognitive Cost-Reference Particle Filtering

Although the CRPF approach can be used in the dynamic system to deal with the uncertainty problem, by controlling the risk and cost and adapting the variance of the noise, it is also limited by the system performance, e.g., the waveform parameter, process noise, SNR, etc. If the iteration can be performed in another dimension, namely in the varying information entropy caused by the varying parameters, through perceiving the exterior environment, the estimation error may be decreased further. To address this challenge, we propose a cognitive cost-reference particle filter algorithm to perform the robust adaptive filter for target tracking.

#### 3.2.1. Cost Function Design

Considering the more complicated feedback in cognitive CRPF (CCRPF), it seems not easy to achieve the closed-form solution of the CRLB even iteratively [46]. Thus, we use the MC approach to approximate the BIM.

Breaking through the limitation of the traditional CRPF method in Equation (25) that only minimizes the cost of particles in a one-time step, we are dedicated to designing a new cost function based on parameter adaption in cognitive radar framework. The processor cost function is defined with a recursive additive structure as follows:

(26) $$C\left(x_{0:k}^{}|z_{1:k}^{},\lambda ;\theta_{k}\right)=\lambda {\hat{C}}_{k}^{}+\Delta C_{k+1}^{}\left(\theta_{k}\right)=\sum_{i=0}^{k}\lambda^{\left(k-i\right)}\Delta C\left(x_{k}^{}|z_{k}^{};\theta_{k}\right).$$

**Lemma** **1.** *Let $\mu_{k,M}$ be the unbiased estimation of mean value $\mu_{k}$, let $\sigma_{k}^{\left(i\right)}\left(\theta_{k}\right)$ be the variance, and let $\Delta C\left(x_{k}^{}|y_{k}^{};\theta_{k}\right)$ be a cost function.*

*If the two following conditions are met:*

*(1) The ${\hat{\sigma }}_{k}^{\left(i\right)}\left(\theta_{k}\right)$ is asymptotically to the $\sigma_{k}^{\left(i\right)}\left(\theta_{k}\right)$, that is*

(27) $$\underset{k\to \infty }{\lim}\left|\sigma_{k}^{\left(i\right)}\left(\theta_{k}\right)-{\hat{\sigma }}_{k}^{\left(i\right)}\left(\theta_{k}\right)\right|=0\;(i.p.)$$

*where i.p. stands for “in probability”.*

*(2) The mean incremental cost $\bar{\Delta C_{k}}=\sum_{i=1}^{M}\omega_{k}^{\left(i\right)}\Delta C\left(x_{k}^{\left(i\right)}|y_{k}^{};\theta_{k}\right)$ converges to the minimal incremental cost*

(28) $$\underset{M\to \infty }{\lim}\left|\Delta C\left(x_{k}^{opt}|y_{k}^{};\theta_{k}\right)-\bar{\Delta C_{k}}\right|=0\;(i.p.),$$

*and then $\mu_{k,M}$ is asymptotically to $\mu_{k}$, so namely the error satisfies*

(29) $$\underset{k\to \infty }{\lim}\left|\mu_{k,M}-\mu_{k}\right|=0\;(i.p.).$$

See Appendix A for a proof.

It can be seen from Equation (26) that the cost function changes with the waveform parameters. According to Lemma 1, we can achieve the asymptotically optimal sequence of state vectors by using the MC approach. The loss function is defined with respect to the cost function of the processor and sensor, modeled by:

(30) $$L_{C,\Theta }\left(\theta_{k}|z_{1:k-1}^{};\Theta_{k-1}\right)\equiv L\left\{C_{k+1}^{}\left(\theta_{k}|z_{1:k-1}^{};\Theta_{k-1}\right),T_{\Theta }\left(\theta_{k}\right)\right\},$$

where $P^{k}$ is the waveform library at time *k*. The loss function should be modeled to balance the predicted conditional Bayes risk/cost and measurement cost. Thus, we define it to be the sum of them, that is,

(31) $$L_{C,\Theta }\left(\theta_{k}|z_{1:k-1}^{};\Theta_{k-1}\right)\equiv C_{k+1}^{}\left(\theta_{k}|z_{1:k-1}^{};\Theta_{k-1}\right)+T_{\Theta }\left(\theta_{k}\right).$$

The next $\theta_{k}^{*}$ is chosen to minimize the loss function, and the choice of the optimal parameter is equivalent to the optimization problem given by:

(32) $$\theta_{k}^{*}=\underset{\theta_{k}^{}\in P}{\arg\min}C_{k+1}^{}\left(\theta_{k}|z_{1:k-1}^{};\Theta_{k-1}\right)\;\mathrm{s.t.}\;\theta_{k}\in \Theta .$$

Standard PF uses the statistical reference, which gives the particles the equal weighting after resampling. CRPF uses the cost reference, which preserves the particle cost after resampling to shift the random grid representation of the cost function toward its local minima. The proposed method is to shift the cost function toward another dimension, namely the vector space effected by the waveform library, to further minimize the cost signal estimates.

#### 3.2.2. Sequential Algorithm and Design Issue

According to Equation (21) and the definition of the risk function, the risk function $R\left(x_{k}^{}|z_{k+1}^{};\theta_{k}\right)$ is a prediction of the cost increment $\Delta C\left(x_{k+1}^{}|z_{k+1};\theta_{k}\right)$, so R(⋅) and ΔC(⋅) are related closely, which can be chosen simply as:

(33) $$\Delta C\left(x_{k}^{}|z_{k};\theta_{k}\right)={\left\|z_{k}-f_{y}\left(x_{k}^{};\theta_{k}\right)\right\|}^{q},$$

(34) $$R\left(x_{k}^{}|z_{k+1}^{};\theta_{k}\right)={\left\|z_{k+1}-f_{y}\left(f_{x}\left(x_{k}^{};\theta_{k}\right)\right)\right\|}^{q}.$$

After resampling, particles are drawn according to the propagation density, in which we add the waveform parameter $\theta_{k}$ because of the cognitive framework, denoted as:

(35) $$x_{k+1}^{\left(i\right)}∼p_{k+1}\left(x_{k+1}^{}|{\hat{x}}_{k}^{\left(i\right)};\theta_{k}\right).$$

The expected E[⋅] with respect to the PDF p(⋅) is used as the constraint of Equation (35), denoted as:

(36) $$E_{p_{k+1}\left(x_{k+1}^{}|x_{k}^{};\theta_{k}\right)}\left[x_{k+1}^{}\right]=f_{x}\left(x_{k}^{};\theta_{k}\right).$$

Therefore, the particles propagate randomly according to the dynamic model, which changes with $\theta_{k}$, that is, the conditional pdf $p_{k+1}\left(x_{k+1}^{}|x_{k}^{}\right)$ still can be arbitrary, but the range of the constraint will be changed by the parameters in another dimension. A zero-mean Gaussian pdf with adaptive variance is used for the particle propagation, denoted as:

(37) $$x_{k+1}^{\left(i\right)}∼N\left(f_{x}\left({\hat{x}}_{k-1}^{\left(i\right)};\theta_{k}\right),\sigma_{k}^{2,\left(i\right)}I_{L_{x}}\right),$$

where $\sigma_{k}^{2,\left(i\right)}$ is the variance, and $I_{L_{x}}$ is the $L_{x}\times L_{x}$ identity function. One choice of adaptive selection for $\sigma_{k}^{2,\left(i\right)}$ is to compute it by the recursive model.

(38) $$\sigma_{k}^{2,\left(i\right)}\left(\theta_{k}\right)=\frac{t-1}{t}\sigma_{k-1}^{2,\left(i\right)}+\frac{{\left\|x_{k}^{\left(i\right)}-f_{x}\left({\hat{x}}_{k-1}^{\left(i\right)};\theta_{k}\right)\right\|}^{2}}{kL_{x}}.$$

In terms of the cost function:

(39) $$C_{k+1}^{\left(i\right)}\left(\theta_{k}\right)=\lambda {\hat{C}}_{k}^{\left(i\right)}+\Delta C_{k+1}^{\left(i\right)}\left(\theta_{k}\right),$$

where $\Delta C_{k+1}^{\left(i\right)}$ is also affected by the newly updated $\theta_{k}$, shown as:

(40) $$\Delta C_{k+1}^{\left(i\right)}\left(\theta_{k}\right)=\Delta C\left(x_{k+1}^{\left(i\right)}|z_{k+1}^{};\theta_{k}\right).$$

The next transmitted waveform is selected. The function μ in Equation (41) is selected to assign large probability masses to lower-cost particles. When we add $\theta_{k}$ to the cost function, the probability masses will be re-assigned as follows:

(41) $${\hat{\pi }}_{k}^{\left(i\right)}\propto \mu_{2}\left\{C_{k}^{\left(i\right)}\left(\theta_{k}\right)\right\}=\frac{1}{{\left(C_{k}^{\left(i\right)}\left(\theta_{k}\right)-\min_{p}\left\{C_{k}^{\left(p\right)}\left(\theta_{k}\right)\right\}+\delta \right)}^{\beta }},$$

where 0<δ<1, β>1. The current state estimation and its prediction covariance matrix is updated by:

(42) $$i_{0}=\arg\max{\hat{\pi }}_{k}^{\left(i\right)},{\hat{x}}_{k}^{\min}={\hat{x}}_{k}^{\left(i_{0}\right)}.$$

We can obtain the optimal sequence of state vectors and the pointwise solution, denoted as:

(43) $${\hat{x}}_{k}^{mean}=\sum_{i=1}^{N_{s}}{\hat{\pi }}_{k}^{\left(i\right)}{\hat{x}}_{k}^{\left(i\right)}.$$

The cognitive structure is combined with the cost-reference function couple, considering the internal and external mechanisms to form the complete CCRPF algorithm. The implementation details are given in Algorithm 3. This can be reduced to the CRPF if using a fixed waveform.
**Algorithm 3.** CCRPF algorithm for maneuvering target tracking problem.**Initialization**, $(\mathrm{for}\;i=1,\ldots ,N_{s}),\;$ 1.    $\mathrm{generate}\;x_{0}^{\left(i\right)}∼p_{0}\left(x_{0}\right)$
$,\;\mathrm{assign}\;\mathrm{the}\;\cos t\;C_{0}^{\left(i\right)}=0$
$,\;\mathrm{and}\;\mathrm{initialize}\;\sigma_{0}^{2,\left(i\right)}.$
**PMF Update** 2.    Start with the initial waveform parameter θ. For each θ compute:
 3.      $\left\{{\hat{x}}_{k-1}^{\left(i\right)},\omega_{k-1}^{\left(i\right)}\right\}=\left\{x_{k-1}^{\left(i\right)},\omega_{k-1}^{\left(i\right)}\right\}$
$,\;i=1,\ldots ,N_{s}$
$,\;z_{k}=f_{y}\left(x_{k}^{},w_{k};\theta \right)$
 4.      $R_{k}^{\left(i\right)}\left(\theta \right)=\lambda C_{k-1}^{\left(i\right)}+{\left\|z_{k}-f_{y}\left(f_{x}\left(x_{k-1}^{\left(i\right)}\right)\right)\right\|}^{q}$
, q=1,2
$;\;i=1,\ldots ,N_{s}$
$,\;\pi_{k}^{\left(i\right)}\propto \mu \left(R_{k}^{\left(i\right)}\right)=\frac{1}{{\left(R_{k}^{\left(i\right)}-\min{\left\{R_{k}^{\left(i\right)}\right\}}_{i=1}^{N_{s}}+\delta \right)}^{\beta }}$
 5.      $\mathrm{Resampling}\;{\hat{x}}_{k-1}^{}={\left\{{\hat{x}}_{k-1}^{\left(i\right)},{\hat{C}}_{k-1}^{\left(i\right)}\right\}}_{i=1}^{N_{s}}$
$\;\mathrm{according}\;\mathrm{to}\;\pi_{k}^{\left(i\right)}$
**Particle Propagation and Waveform Selection** 6.      
$x_{k}^{\left(i\right)}∼p_{k}\left(x_{k}^{}|{\hat{x}}_{k-1}^{\left(i\right)}\right)$
$,\;\mathrm{compute}\;\mathrm{the}\;\mathrm{cost}\;C_{k}^{\left(i\right)}\left(\theta \right)=\lambda C_{k-1}^{\left(i\right)}+{\left\|z_{k}-f_{y}\left(x_{k}^{\left(i\right)}\right)\right\|}^{q}$
 7.      $\mathrm{Compute}\;\mathrm{the}\;\cos t\;\mathrm{function}\;\theta_{k}^{*}=\underset{\theta }{\arg\min}C_{\Theta }^{\left(i\right)}\left(\theta_{k}|z_{1:k-1}^{};\Theta_{k-1}\right)+C_{\Theta }\left(\theta_{k}\right)$
 8.    $\mathrm{Select}\;\mathrm{the}\;\mathrm{optimal}\;\mathrm{waveform}\;\mathrm{parameter}\;\theta_{k}=\theta_{k}^{*}$
**Particle and Measurement Recursive Update** 9.      ${\hat{x}}_{k}^{\left(i\right)}={\hat{x}}_{k}^{\left(i\right)}$
$,\;z_{k}=f_{y}\left(x_{k}^{},w_{k};\theta_{k}\right)$
$,\;C_{k}^{\left(i\right)}=C_{k}^{\left(i\right)}\left(\theta_{k}\right)$
 10.      $\sigma_{k}^{2,\left(i\right)}\left(\theta_{k}\right)=\left\{ \begin{array}{ll} \sigma_{k-1}^{2,\left(i\right)} & t\leq 10\\ \frac{k-1}{k}\sigma_{k-1}^{2,\left(i\right)}+\frac{{\left\|x_{k}^{\left(i\right)}-f_{x}\left({\hat{x}}_{k-1}^{\left(i\right)}\right)\right\|}^{2}}{k\times \dim\left[x\right]} & t>10 \end{array}\right.$, *i* = 1, …, *N_s_***Information Update and State Estimation** 11.    ${\hat{\pi }}_{k}^{\left(i\right)}\propto \mu_{2}\left(C_{k}^{\left(i\right)};\theta_{k}\right)=\frac{1}{{\left(C_{k}^{\left(i\right)}-\min{\left\{C_{k}^{\left(i\right)}\right\}}_{i=1}^{N_{s}}+\delta \right)}^{\beta }},$ where α,β>0. Normalize the PMF.
 12.      
${\hat{x}}_{k}={\hat{x}}_{k}^{mean}=\sum_{i=1}^{N_{s}}{\hat{\pi }}_{k}^{\left(i\right)}{\hat{x}}_{k}^{\left(i\right)}$
$.\;\mathrm{Save}\;\mathrm{the}\;{\hat{x}}_{k}$
$\;\mathrm{and}\;{\hat{P}}_{k}^{}\left(\theta_{k}\right).$


### 3.3. Convergence of CCRPF Algorithm

Due to the change of waveform parameter, resulting in the change of the measurement noise, convergence results regarding CRPFs may be not valid for the proposed algorithm. We assess the convergence. More details about the preliminary definitions and the derivation of the conditions are referred to in [26].

**Lemma** **2.** *Let $\Delta C\left(x_{k}^{}|y_{k}^{}\right)$ be a cost function. With a fixed waveform parameter θ, if the three following conditions are met:*

*(1) The set function $\mu_{k}\left(A\subseteq {\left\{x_{k}^{\left(i\right)}\right\}}_{i=1}^{M}\right)=\sum_{x\in A}^{}\mu \left(\Delta C\left(x_{k}|y_{k}^{}\right)\right)$ satisfies*

(44) $$\underset{M\to \infty }{\lim}\mathrm{Pr}\left[1-\frac{\mu_{k}\left(S^{M}\left(x_{k}^{opt}\left(\theta \right),\varepsilon \right)\right)}{\mu_{k}\left({\left\{x_{k}^{\left(i\right)}\left(\theta \right)\right\}}_{i=1}^{M}\right)}\geq \delta \right]=0\;\forall \delta >0,$$

*where Pr[⋅] denotes probability, ${\left\{x_{k}^{\left(i\right)}\right\}}_{i=1}^{M}$ is a set of particles drawn at time step k, and $S^{M}\left\{x_{k}^{opt}\left(\theta \right),\varepsilon \right\}=\left\{x\in {\left\{x_{k}^{\left(i\right)}\right\}}_{i=1}^{M}:\left\|x-x_{k}^{opt}\right\|<\varepsilon ;\theta \right\}$.*

*(2) The mean incremental cost $\bar{\Delta C_{k}}=\sum_{i=1}^{M}\omega_{k}^{\left(i\right)}\Delta C\left(x_{k}^{\left(i\right)}|y_{k}^{}\right)$ converges to the minimal incremental cost*

(45) $$\underset{M\to \infty }{\lim}\left|\Delta C\left(x_{k}^{opt}|y_{k}^{};\theta \right)-\bar{\Delta C_{k}}\right|=0\;(i.p.).$$

*(3) The mean cost estimate is asymptotically optimal,*

(46) $$\underset{M\to \infty }{\lim}\left|\Delta C\left({\tilde{x}}_{k}^{mean}|y_{k}^{};\theta \right)-\Delta C\left(x_{k}^{opt}|y_{k}^{};\theta \right)\right|=0\;(i.p.),$$

*where*${\tilde{x}}_{k}^{mean}=\sum_{i=1}^{M}\pi_{k}^{\left(i\right)}x_{k}^{\left(i\right)}$, *then with the adaptive parameter $\theta_{k}$, the set function satisfies:*

(47) $$\underset{M\to \infty }{\lim}\left[\mu_{k}\left(S^{M}\left(x_{k}^{opt}\left(\theta_{k}\right),\varepsilon \right)\right)/\mu_{k}\left({\left\{x_{k}^{\left(i\right)}\left(\theta_{k}\right)\right\}}_{i=1}^{M}\right)\right]=1\;(i.p.),$$

*and the mean cost estimate is asymptotically optimal, that is:*

(48) $$\underset{M\to \infty }{\lim}\left|\Delta C\left({\tilde{x}}_{k}^{mean}|y_{k}^{};\theta_{k}\right)-\Delta C\left(x_{k}^{opt}|y_{k}^{};\theta_{k}\right)\right|=0\;(i.p.).$$

See Appendix B for a proof.

## 4. Numerical Results and Discussion

### 4.1. Dynamic Model

The evolution of the target state and the corresponding measurements are described by a known discrete-time stochastic model separately [47]. Consider the target moving in the x−y plane according to the model:

(49) $$x_{k+1}=F_{k}x_{k}+v_{k},$$

where ${\left[x_{1,k}^{},x_{2,k}^{},x_{3,k}^{},x_{4,k}^{}\right]}^{T}$ denotes the state vector **X***_k_* of the target, $\left(x_{1,k}^{}x_{2,k}^{}\right)$, and $\left(x_{3,k}^{},x_{4,k}^{}\right)$ denotes the position and the velocity, respectively. $v_{k}={\left[0\;0\;v_{x}\;v_{y}\right]}^{T}$ is the system noise. We assume that the sampling period is short enough for the velocity to be a constant during the period, so the state transition matrix is defined as:

(50) $$F_{k}=\left[\begin{array}{ll} F_{11} & F_{12}\\ 0 & F_{22} \end{array}\right],F_{11}=F_{22}=\left[\begin{array}{l} 1\;0\\ 0\;1 \end{array}\right],F_{12}=\left[\begin{array}{l} T\;0\\ 0\;T \end{array}\right].$$

The measurement equation is considered to be highly nonlinear, and as described as follows, $z_{k}={\left[r_{k},{\dot{r}}_{k},\beta_{k}\right]}^{T}$ is selected as the observable vector, and the observation matrix is denoted as:

(51) $$z_{k}={\left[r_{k},\;{\dot{r}}_{k},\;\beta_{k}\right]}^{T}+w_{k},$$

where the range $r_{k}$, range-rate ${\dot{r}}_{k}$, and bearing $\beta_{k}$ compose the observable vector, that is:

(52) $$r_{k}={\left(x_{1,k}^{2}+x_{2,k}^{2}\right)}^{1/2},\;{\dot{r}}_{k}={\left(x_{3,k}^{2}+x_{4,k}^{2}\right)}^{1/2},\;\beta_{k}=\arctan\left(x_{2,k}^{}/x_{1,k}^{}\right).$$

Select Equation (15) to be the model of $R_{k}$. The fixed-waveform parameter is set as $\lambda_{0}=50\times 10^{-6}s$, $b_{0}=60\times 10^{9}\mathrm{yad}/s^{2}$, and the initial SNR is set as $\eta_{0}=16$, so $R_{0}=R\left(\lambda_{0},b_{0},\eta_{0}\right)$. The initial true state of the target is ${\left[10,\;-5,\;-0.2,\;0.2\right]}^{T}$, and the given initial state is set as ${\left[6\;-2\;0\;0\right]}^{T}$, $\sigma_{0}^{2,\left(i\right)}={\left[1.5\;10\;0.1\;1.5\right]}^{T}$. The sampling period is T=1 s. The generally used heavy-tailed distributions include Laplace distribution, t distribution, uniform distribution, and Gaussian distribution with large variance. Considering that the mixture Gaussian is applied, the sum of the different weighted Gaussian noises with different parameters can be used as the modeling of flicker noise $w_{k}\left(\theta_{k-1}\right)$. The pdf of flicker noise can be denoted as:

(53) $$v_{x,y}∼0.1N\left(0,1\right)+4\times 10^{-4}N\left(0,1\right)+10^{-6}N\left(0,1\right),$$

(54) $$w_{r}∼\sqrt{0.1}N\left(0,\sigma_{r}\right)+0.04\times 10^{-4}N\left(0,\sigma_{r}\right)+2.5\times 10^{-5}N\left(0,\sigma_{r}\right),$$

(55) $$w_{\beta }∼0.1N\left(0,\sigma_{\beta }\right)+4\times 10^{-4}N\left(0,\sigma_{\beta }\right)+1.6\times 10^{-7}N\left(0,\sigma_{\beta }\right),$$

where $N\left(\mu_{i},\Sigma_{i}\right)$ denotes the Gaussian distribution with the mean of $\mu_{i}$ and the variance of $\Sigma_{i}$. The particle number is 400.

For a particular scenario and parameters, the overall performance of a filter is evaluated using the metric that is the ARMSE. We use it to estimate the tracking accuracy of the algorithms, and it is defined as:

(56) $$\mathrm{ARMSE}=\sqrt{\frac{1}{N_{m}K}\sum_{i=1}^{N_{m}}\sum_{k=1}^{K}\left[{\left({\hat{x}}_{1,k}^{i}-x_{1,k}\right)}^{2}+{\left({\hat{x}}_{3,k}^{i}-x_{3,k}\right)}^{2}\right]},$$

where K is the total number of time steps and $N_{m}$ is the total number of independent MC runs. Let $\left(x_{1,k},x_{3,k}\right)$ and $\left({\hat{x}}_{1,k}^{i},{\hat{x}}_{3,k}^{i}\right)$ denote the true and the estimated target position at time k at the i-th MC run, respectively. Then, the RMSE of the estimated states at k can be computed as [48]:

(57) $$\mathrm{RMSE}\left(k\right)=\sqrt{\frac{1}{N_{m}}\sum_{i=1}^{N_{m}}\left[{\left({\hat{x}}_{1,k}^{i}-x_{1,k}\right)}^{2}+{\left({\hat{x}}_{2,k}^{i}-x_{2,k}\right)}^{2}\right]}.$$

The upper and lower limits of the waveform parameters are determined according to the transmitter specifications, and the waveform library can be obtained as:

(58) $$P=\left\{\lambda \in \left[\lambda_{\min}:\Delta \lambda :\lambda_{\max}\right],b\in \left[b_{\min}:\Delta b:b_{\max}\right]\right\},$$

where Δ*λ* denotes the step-size of the envelope duration, and Δ*b* denotes the step-size of the chirp rate.

### 4.2. Selection of Function μ and Parameters δ

Before implementing the simulation experiments, we check the behavior of the proposed method with different function μ and different values of tuning parameter δ in Equations (33), (34) and (41), to select suitable μ and δ. 

**Figure 3:**
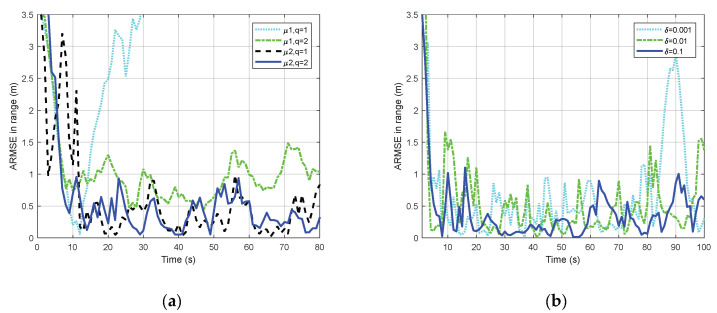
Selection of functions and parameters: (**a**) Mean absolute deviation for different μ functions; (**b**) Mean absolute deviation for different δ values.

The selection of the tuning parameters is investigated in Figure 3. Figure 3a shows four cases of mean absolute deviation after the tracking, with varying values set of μ and q. The performance in the case (μ1, q=1) seems well at beginning but shows a rapid degradation soon. The performances of the other cases are similar, wherein case (μ2, q=2) shows a slightly better overall performance. Three cases with varying values of δ are considered in Figure 3b. It is clear that a small δ may result in a worse improvement. Therefore, it is reasonable to select μ2, q=2, δ=1 for the rest of the simulations according to the analysis and conclusion.

### 4.3. Simulation Results 

#### 4.3.1. Scenario 1: Unknown Statistics in General Environment

We assume that the process noise and measurement noise is independent and temporally white in this case, with the mixture Gaussian pdf as Equations (53)–(55) show, but the given methods are mismatched with the dynamic systems and models.

**Figure 4:**
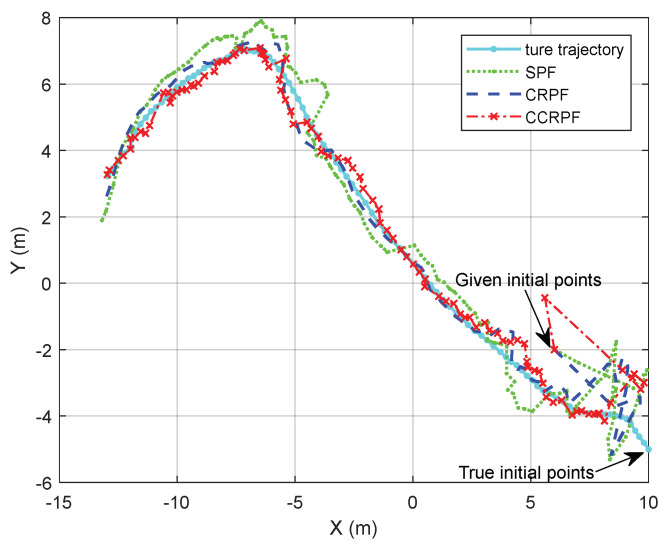
Tracking example.

We apply SPF, CRPF, and CCRPF for target tracking in a two-dimensional space for performance assessment. Figure 4 displays the true path against the tracks of these filters. The other two tracks the target with an error smaller than the SPF, wherein the CCRPF outperforms CRPF slightly. Note how the trajectory of CCRPF deviates from the true path slightly due to the cost function and cognitive structure. EKF does not show the performance of robustness, and we do not show the result here.

**Figure 5:**
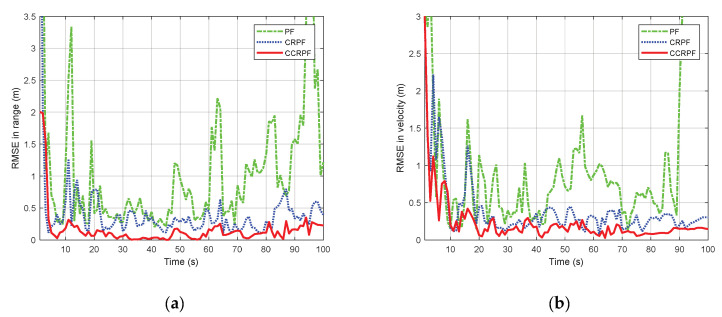
Comparison of root mean square error (RMSE) between cognitive CRPF (CCRPF) with PF and CRPF: (**a**) estimated position; (**b**) Estimated velocity.

All error curves corresponding to the above three filters were obtained by simulation runs. The results for the estimation error of the range and range-rate are shown in Figure 5a,b respectively. Observe from Figure 5 that the SPF introduces large bias in the estimation. CRPF and CCRPF are unbiased throughout the observation period. As for the comparison of CRPF and CCRPF, the overall performance of CCRPF is considered better than that of CRPF. Particularly, the CCRPF estimation is approaching zero bias except for the very beginning stage, indicating a fairly robust performance.

In term of the convergence speed, the convergence of CRPF and CCRPF shows more rapidly than SPF and keeps stable; thus, they are more robust than SPF in tracking performance. The CRPF converges the error asymptotically, while the proposed method achieves convergence in less than 10 time steps, due to the real-time perception of environment in the cognitive algorithm.

CRPF does not have a bad overall performance, but it provides higher peak errors than CCRPF since the cost reference mechanism can only use the fixed waveform, that is to say, it can only be adaptive in the constant environment conceived by the fixed waveform. However, cognitive radar based on CCRPF can not only perceive the dynamic environment by the dynamic waveform, but can also stay adaptive during the recursion after the waveform is chosen. Thus, CCRPF can suppresses the peak error on the fly and limits the overall magnitude of the error, showing the superiority. 

A summary of these results and the detailed comparison are also tabulated in Table 1. The parameter of fixed waveform 1 is $\lambda =10\times 10^{-6}$, $b=10\times 10^{9}$, and the parameter of fixed waveform 2 is $\lambda =50\times 10^{-6}$, $b=60\times 10^{9}$. The first row of the table lists the metrics corresponding to the ranging performance by MC simulations with 40 independent runs, which is computed using Equation (56). The second row gives the performance on estimation of range-rate.

From this table, more concretely, we see that the SPF shows a degradation in performance, it has the lowest tracking accuracy of the compared methods. From the comparison of PFs using two different fixed waveforms, we find that PF with waveform 2 is better than that with waveform 1, due to the effects of the measurement noise obtained by different waveforms, and the same conclusion can be found in CRPFs. It is clear that the best filters for this case was CCRPF, which achieves an excellent performance of a 15% improvement over the SPF-2 and a 3% improvement over the CRPF-2.

The unknown environment has a huge influence on SPF for tracking. The mismatch of the noise is the main cause result in the performance degradation. CRPF is less affected since it takes advantage of the cost reference mechanism, so it has better tracking accuracy than SPF. The mastery of environmental information in time has an extra contribution in the proposed algorithm, which has more obvious performance improvement compared with others because of the robust module working cooperatively with the cognitive structure. This also indicates that it is a good supplement to the cognitive radar tracking approach.

**Figure 6:**
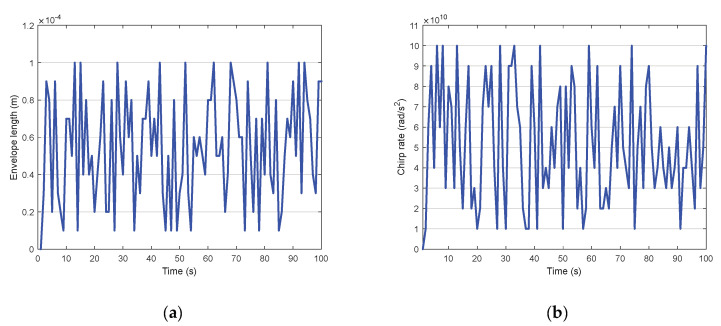
Waveform selection across time: (**a**) On chip rate; (**b**) On length of pulse envelope.

To study how the cognition process evolves across time, we have plotted the waveform selection for both the chirp rate and the duration of the pulse envelope in Figure 6a,b. We observe that the transition of the chirp rate is switched from maximum up-sweep to maximum down-sweep.

#### 4.3.2. Scenario 2: Unknown Statistics in Dynamic Noise Environment

Unknown and abrupt change of noise would be considered in this case. The main parameters are the same with Scenario 1. Measurement noise covariance R is known a priori, and the actual process noise covariance Q is set as:

(59) $$\left\{ \begin{array}{ll} Q_{0}=Q^{\circ }, & k=1,\ldots ,60\\ Q_{0}=80\times Q^{\circ }, & k=61,\ldots ,80 \end{array}\right..$$

**Figure 7:**
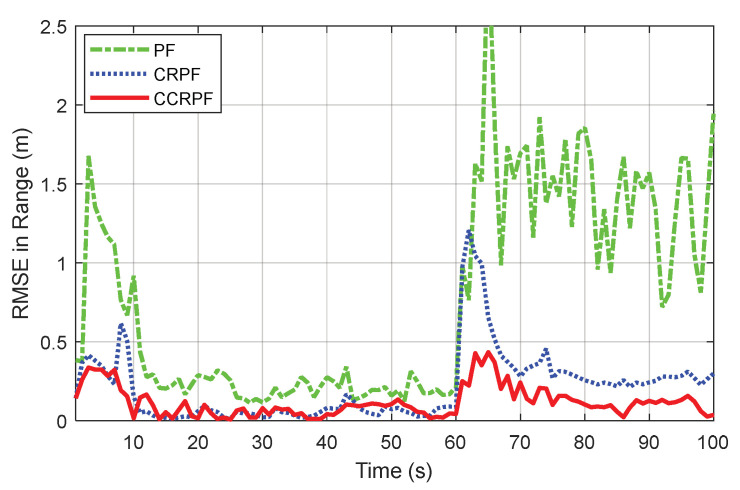
RMSE of the estimated range.

Figure 7 shows the RMSE curves corresponding to the four filters. When k=1,…,60 with a priori known noise, all algorithms can track the target successfully. In the subsequent phase when Q is increasing from the time step 60 abruptly, although SPF could not be seen as a failure to track the target, its convergence effect and speed are all the worst. CRPF shows a similar convergence speed to CCRPF, because of the similar robust module, but its overall performance during the dynamic noise stage is obviously inferior to the latter. The proposed approach shows the superiority again, with the lowest error and fastest convergence speed.

A summary of the results and the detailed comparison are also tabulated in Table 2. We do not use a different waveform in SPF and CRPF anymore because similar work has already been done in Scenario 1.

## 5. Conclusions

We have concentrated on the tracking method of the cognitive radar based on particle filter. The CCRPF for cognitive radar is proposed as a significant step toward random dynamic systems with unknown statistics. In this work, the mathematical model of cognitive radar has been derived, CRLB and the corresponding cost function have been designed, the cognitive PF algorithm has been presented by completely reconstructing the propagation and update process, and the proposed algorithm has been developed by updating the cost function and noise variance. Moreover, the convergence of the approach has been proofed. The simulation results illustrate that the state error prediction is more adjacent to the CRLB, and the proposed method showed a good outperforming result over the existing methods in accuracy and robustness on highly nonlinear dynamic systems with unknown statistics or a complicated environment. The application of cognitive radar and the adaptation of a traditional filter have been expanded to a wider scope.

Future work: (1) PF is approximately globally optimal, while cognitive estimation is biased, so the closed-form of the optimal solution is not easy to obtain in the PF-based cognitive tracking method, and we can only use MC to get the solution or proof the convergence. However, the cost function in CRPF might have the optimal solution; thus, the cost function design is deserves to have further study. (2) Using novel SMC methods as the cognitive framework may have better prospects in dealing with dynamic system problems with unknown or uncertain statistics. (3) The lower limit and dynamic change of particle number could also be determined.

## Figures and Tables

**Table 1:** Performance comparison of algorithms.

ARMSE	SPF-1 ^1^	SPF-2 ^2^	CRPF-1 ^3^	CRPF-2 ^4^	CCRPF
**Range**	1.54	1.09	1.26	0.96	0.93
**Range-rate**	0.37	0.24	0.17	0.16	0.14

^1^ SPF-1: SPF with fixed waveform 1, ^2^ SPF-2: SPF with fixed waveform 2, ^3^ CRPF-1: CRPF with fixed waveform 1, ^4^ CRPF-2: CRPF with fixed waveform 2.

**Table 2:** Performance comparison of algorithms.

ARMSE	SPF	CRPF	CCRPF
k≤30	0.51	0.58	0.23
k>30	1.49	0.20	0.19

## Abbreviations

The following abbreviations are used in this paper:

PF	Particle Filtering
MC	Monte Carlo
PMCMC	Particle Markov Chain Monte Carlo
SMC	Sequential Monte Carlo
SMC^2^	Sequential Monte Carlo Square
SIS	Sequential Importance Sampling
SIR	Sampling Importance Resampling
APF	Adaptive PF
CRLB	Cramér–Rao Lower Bound
CPF	Cognitive PF
SPF	Standard PF
MSE	Mean Square Error
PAC	Perception-action Cycle
BCRB	Bayesian Cramér–Rao lower bound
BIM	Bayesian Fisher Information Matrix
MMSE	Minimum MSE
SNR	Signal-to-noise Ratio
LFM	Linear Frequency Modulation
RMSE	Root Mean Square Error
PCRB	Posterior Cramér–Rao Bound
CRPF	Cost-reference Particle Filter
WPS	Weighted-particle Set
PMF	Probability Mass Function
CCRPF	Cognitive Cost-reference Particle Filter
PDF	Probability Density Function
ARMSE	Average Root Mean Square Error
FIM	Fisher Information Matrix
KLD	Kullback–Leibler Distance
GRNN	General Regression Neural Network
CKF	Cubature Kalman Filter
CD-CKF	Continuous-Discrete Cubature Kalman Filter

## Appendix A

In the MC integration, Equation (1) is as the expected value of $f_{k}\left(x_{i}^{}\right)$, so it can be estimated as the sample mean:

(A1) $$\mu_{k,M}=\frac{1}{M}\sum_{i=1}^{M}f_{k}\left(x_{i}^{}\right).$$

Supposing $\mu_{k}=E\left[f_{k}\left(x\right)\right]$, it can be proved that $\mu_{k,M}$ is the unbiased estimation of $\mu_{k}$, and the non-zero bounded variance can be denoted as:

(A2) $$\sigma_{k}^{2}\left(\theta_{k}\right)=\int_{D}{\left(f_{k}\left(x\right)-\mu_{k}\right)}^{2}p\left(x\right)dx<+\infty .$$

Given the confidence level 1−α, the error can be denoted as:

(A3) $$\left|\mu_{k,M}-\mu_{k}\right|\leq \lambda_{\alpha }{\hat{\sigma }}_{k}^{}\left(\theta_{k}\right)/\sqrt{M}.$$

Owing to Condition (1), we can obtain the expression:

(A4) $$\underset{M\to \infty }{\lim}\left|\sigma_{k}\left(\theta_{k}\right)-{\hat{\sigma }}_{k}^{}\left(\theta_{k}\right)\right|=0\;(i.p.),$$

(A5) $$\underset{\begin{array}{l} M\to \infty \\ k\to \infty \end{array}}{\lim}\left|\mu_{k,M}-\mu_{k}\right|=0\;(i.p.).$$

## Appendix B

When we choose the optimal parameter $\theta_{k}$, and from the preliminary definitions we can exploit that:

(A6) $$\underset{M\to \infty }{\lim}E_{\mathrm{Pr}\left[n_{M}\right]}\left[n_{M}\right]=\left(1-\gamma \right)\underset{M\to \infty }{\lim}M\;(i.p.),$$

then the following relationship holds:

(A7) $$\begin{array}{ll} \underset{M\to \infty }{\lim} & \frac{E_{\mathrm{Pr}\left[n_{M}\right]}\left[n_{M}\right]}{\mu_{k}\left(S^{M}\left(x_{k}^{opt},\varepsilon ;\theta_{k}\right)\right)}\\ & =\left(1-\gamma \right)\underset{M\to \infty }{\lim}\frac{M}{\mu_{k}\left(S^{M}\left(x_{k}^{opt},\varepsilon ;\theta_{k}\right)\right)}\\ & \leq \left(1-\gamma \right)\underset{M\to \infty }{\lim}\frac{M}{S_{in}}=0 \end{array}$$

Thus, we can write:

(A8) $$\underset{M\to \infty }{\lim}\mathrm{Pr}\left[1-\frac{\mu_{k}\left(S^{M}\left(x_{k}^{opt},\varepsilon ;\theta_{k}\right)\right)}{\mu_{k}\left({\left\{x_{k}^{\left(i\right)}\right\}}_{i=1}^{M}\right)}\geq \delta \right]≅\frac{1-\delta }{\delta }S_{out}\underset{M\to \infty }{\lim}\frac{E_{\mathrm{Pr}\left[n_{M}\right]}\left[n_{M}\right]}{\mu_{k}\left(S^{M}\left(x_{k}^{opt},\varepsilon ;\theta_{k}\right)\right)}=0,$$

(A9) $$\underset{M\to \infty }{\lim}\left[\mu_{k}\left(S^{M}\left(x_{k}^{opt}\left(\theta_{k}\right),\varepsilon \right)\right)/\mu_{k}\left({\left\{x_{k}^{\left(i\right)}\left(\theta_{k}\right)\right\}}_{i=1}^{M}\right)\right]=1\;(i.p.).$$

The proof of asymptotically optimization of the mean cost estimate is carried out by following steps:

(A10) $$\begin{array}{ll} \underset{M\to \infty }{\lim} & \left|\Delta C\left({\tilde{x}}_{k}^{mean}|y_{k}^{};\theta_{k}\right)-\Delta C\left(x_{k}^{opt}|y_{k}^{};\theta_{k}\right)\right|\\ & \leq \underset{M\to \infty }{\lim}\left|\underset{x_{k}^{}\in S^{M}\left(x_{k}^{opt},\varepsilon \right)}{\sup}\;\Delta C\left(x_{k}^{}|y_{k}^{};\theta \right)-\Delta C\left(x_{k}^{opt}|y_{k}^{};\theta \right)\right|\\ & =B\left(\varepsilon =1/\sqrt{M}\right)=0 \end{array}$$
